# Removal of evolutionarily conserved functional MYC domains in a tilapia cell line using a vector-based CRISPR/Cas9 system

**DOI:** 10.1038/s41598-023-37928-x

**Published:** 2023-07-26

**Authors:** Chanhee Kim, Avner Cnaani, Dietmar Kültz

**Affiliations:** 1 Department of Animal Sciences, University of California, Davis, CA 95616 USA; 2 Department of Poultry and Aquaculture, Institute of Animal Sciences, Agricultural Research Organization, Volcani Center, 7528809 Rishon LeZion, Israel

**Keywords:** Genetic engineering, Ichthyology

## Abstract

MYC transcription factors have critical roles in facilitating a variety of cellular functions that have been highly conserved among species during evolution. However, despite circumstantial evidence for an involvement of MYC in animal osmoregulation, mechanistic links between MYC function and osmoregulation are missing. Mozambique tilapia (*Oreochromis mossambicus*) represents an excellent model system to study these links because it is highly euryhaline and highly tolerant to osmotic (salinity) stress at both the whole organism and cellular levels of biological organization. Here, we utilize an *O. mossambicus* brain cell line and an optimized vector-based CRISPR/Cas9 system to functionally disrupt MYC in the tilapia genome and to establish causal links between MYC and cell functions, including cellular osmoregulation. A cell isolation and dilution strategy yielded polyclonal *myca* (a gene encoding MYC) knockout (ko) cell pools with low genetic variability and high gene editing efficiencies (as high as 98.2%). Subsequent isolation and dilution of cells from these pools produced a *myca* ko cell line harboring a 1-bp deletion that caused a frameshift mutation. This frameshift functionally inactivated the transcriptional regulatory and DNA-binding domains predicted by bioinformatics and structural analyses. Both the polyclonal and monoclonal *myca* ko cell lines were viable, propagated well in standard medium, and differed from wild-type cells in morphology. As such, they represent a new tool for causally linking *myca* to cellular osmoregulation and other cell functions.

## Introduction

MYC family genes encode class III basic helix-turn-helix (bHLH) transcription factors (MYC TF) that have been evolutionarily conserved in many animal species for at least 400 million years^1,2^. They are known to have canonical roles in cell proliferation, differentiation, and apoptosis and are characterized by the presence of a leucine zipper (LZ) adjacent to the bHLH domain^1,3^. The bHLH and LZ domains are common functional motifs for DNA binding and dimerization that are often also found in other TF families, which indicates that these domains arose from an evolutionarily ancient ancestral TF. MYC TF also harbors a less common motif, a transcriptional regulatory domain, that is less evolutionarily conserved across species than the DNA-binding and dimerization domains^4^. This transcriptional regulatory domain was shown to strongly control transformation of rat embryo fibroblasts, as evidenced by using a domain deletion mutant^5^. Moreover, it has been suggested that deletion of any of the conserved domains outlined above can significantly impair MYC function^6^. Several lines of indirect evidence suggest that MYC TF is involved in governing cellular osmoregulation^7^. For example, a recent study has revealed that the *myo*-inositol biosynthesis (MIB) pathway of euryhaline turbot (*Scophthalmus maximus*) is positively regulated by MYC, which was demonstrated using an RNAi-mediated knockdown approach^8^. MYC has also been reported to directly modulate responses to abiotic stressors, including salinity stress, in plants (e.g., *Arabidopsis thaliana*^9,10^). This modulation is mediated via key hormonal signaling pathways important for plant salinity tolerance^11^. Such salinity stress-induced, non-canonical roles of MYC TF in plants were also indicated by an *in-silico* prediction study using bread wheat (*Triticum aestivum*)^12^. Moreover, microarray analyses of MYC ko rat cell lines revealed inositol monophosphatase (IMPA, A2 isoform), a key enzyme in the *myo*-inositol biosynthesis (MIB) compatible osmolyte pathway, as a target gene of MYC^13^. These studies suggest that MYC TF is important for controlling osmoregulatory mechanisms in eukaryotes.

The recent revolution of gene targeting approaches by implementing CRISPR/Cas-based approaches has enabled highly accurate and efficient genome editing that is superior to older gene targeting methods such as TALENs or ZFNs, which require covalent linkage of a specific DNA binding domain to a nuclease^14–16^. This innovative system was initially adopted from bacteria and archaea, in which it had evolved as a pathogen nucleic acid-targeting defense mechanism that conferred resistance to viral infection^17,18^. The simplicity and high efficiency of the CRISPR/Cas9 system renders it convenient, cost-effective, and multimodal tool for gene editing in a variety of organisms^19^. Most studies using this system to date have focused on model organisms such as human cell lines^20^ and mouse^21^ both in vivo and in vitro*.* They have demonstrated the great power of knockout (ko) models for functional studies aimed at causally linking genotypes and phenotypes^22–24^. In contrast to the well-established mammalian models, CRISPR/Cas9 approaches have been used to a lesser extent with lower vertebrates such as fish, even though numerous studies have shown that this gene targeting system can be successfully utilized to genetically modify aquaculture fish species in vivo^25–28^. For instance, double-allelic ko mutations were introduced in Atlantic salmon (*Salmo salar*) to alter pigmentation^25^, Insulin-like Growth Factor Binding Protein-2b (IGF-BP2b) was targeted for ko in rainbow trout (*Oncorhynchus mykiss*)^26^, alligator cathelicidin gene was targeted in a non-coding region of channel catfish (*Ictalurus punctatus*) genome (Simora et al. 2020) and myostatin ko mutations were performed in Channel catfish (Ictalurus punctatus) to improve growth and disease resistance (Coogan et al., 2022). Examples for in vivo gene targeting in fish also include zebrafish (*Danio rerio*) where all-in-one CRISPR/Cas9 components were injected into fertilized one-cell stage embryos to generate ko mutants^28^.

Compared to a considerable number of CRISPR/Cas9 studies in whole fish in vivo, gene editing of fish cell lines in vitro has been rarely used and remains to be explored as a promising tool for high-throughput and low-cost establishment of causality between cellular phenotypes and genomic loci of interest. The first fish cell line that was genetically modified by CRISPR/Cas9 technology was reported for Chinook salmon (*Oncorhynchus tshawytscha*)^29^. Chinook salmon cell lines were also used to demonstrate the functionality of a vector-based expression system^30^, as well as to optimize lentivirus-mediated infection for efficient delivery of recombinant DNA into host cells^31^. Our lab has recently optimized and established a vector-based CRISPR/Cas9 platform for tilapia cell lines^32^. This study was the first to enable in vitro gene targeting in euryhaline tilapia cells. Our vector-based in vitro approach differs from the in vivo approach used for whole tilapia, which is based on microinjection of gRNA and Cas9 mRNA or protein into Nile tilapia (*Oreochromis niloticus*) fertilized eggs^33^.

Recent CRISPR/Cas9 approaches aim to obtain highly precise and consistent ko models that are characterized by very high gene editing efficiency and/or clonality, to exclude potentially confounding factors arising from heterogeneity of ko cells^34–36^. Although highly heterogeneous, pooled ko cell populations with high mutational efficiency (about 80% or above) are routinely used for short-term loss-of-function studies, interference arising from expression of wild-type or variable mutant proteins remains a concern^37^. Therefore, recent efforts have been geared towards generating homozygous clonal ko cell models, to ensure that the protein function is completely disrupted and that the resulting mutant proteins that cause effects, are consistent. Several previous studies have reported successful production of clonal ko cell lines for some fish species. For example, Liu et al. have generated a Japanese medaka (*Oryzias latipes*) ko cell line using RNP transfection for CRISPR/Cas9 gene targeting, followed by isolating a ko clonal cell line having a 9-nt deletion in the *sytl5* gene starting with an initial ko cell pool showing 50% gene editing efficiency^38^. Furthermore, a modified Chinook salmon (*Oncorhynchus tshawytscha*) cell line that stably expresses Cas9 protein has been used to generate a monoclonal *stat2* ko cell line harboring a 2-nt frame-shift deletion in *stat2*^39^. Such recent efforts to either generate edited pools (polyclonal) or isolate clonal lines (monoclonal) of CRIPSR/Cas9 gene-edited cells can be expanded beyond canonical model species of fish to enable broad comparative and evolutionary studies^40,41^.

In this study, genetically engineered polyclonal and monoclonal tilapia cell lines were generated to facilitate studies of cellular functions of MYC TF, specifically its role in osmoregulation. A strategy utilizing a DNA vector-based CRISPR/Cas9 system followed by serial dilution of mutant cells for efficiently isolating clonal ko cell lines has been applied. We present the first successful report of applying targeted gene-editing in combination with serial dilution of a heterogeneous cell population to generate low genetic variability polyclonal and monoclonal tilapia cell lines to enable future functional analyses for assessment of causal genotype–phenotype links.

## Materials and methods

### Cell culture

A tilapia Cas9-OmB cell line^32^ previously generated in our lab was used in this study. The genomic presence and expression of Cas9 transgene was verified by an array of PCRs targeting transgene amplicons using both genomic DNA (gDNA) and complementary DNA (cDNA). Cas9-OmB cell working stock (passage 40 of the original OmB cell line^42^; P40) was thawed and maintained at ambient CO_2_ and 26 °C in L-15 medium (Hyclone, SH30910.03) containing 10% (vol/vol) fetal bovine serum (FBS, Gibco, 11415-064), 1% (vol/vol) Penicillin–Streptomycin (Sigma-Aldrich, P4333). When culture plates reached a confluency of 80–90%, cells were passaged (at 3–4-day intervals) using a 1:5 splitting ratio. For applying hyperosmotic stress to cells, hyperosmotic (650 mOsmol/kg) was prepared using hypersaline stock solution (osmolality: 2,820 mOsmol/kg). This stock solution was made by adding an appropriate amount of sodium chloride (NaCl) to regular isosmotic (310 mOsmol/kg) L-15 medium. The hypersaline stock solution was then diluted with isosmotic medium to obtain hyperosmotic medium of 650 mOsmol/kg. Medium osmolality was always confirmed using a freezing point micro-osmometer (Advanced Instruments).

### Generation of sgRNA vectors

The *O. niloticus* reference genome deposited at NCBI was used to derive the coding sequence (CDS) for tilapia *myca*, the MYC proto-oncogene bHLH transcription factor a (Gene ID: 100689989). A *myca* CDS region spanning exons 2 and 3 was submitted to CRISPOR^43^ version 5.01 to design small guide RNAs (sgRNAs) for efficient gene targeting of *myca*. In addition to using CRISPOR, the tilapia *myca* gene was also analyzed with the CRISPR Knockout Guide Design tool provided by SYNTHEGO (https://design.synthego.com/#/) to design sgRNAs using different algorithms. CRISPOR and SYNTHEGO tools both support convenient tilapia gRNA design by providing an integrated reference genome of *O. niloticus* (Ensembl 76-Orenil1.0) for calculating off-target effect of sgRNAs by comparison to the whole genome sequences. This aspect of sgRNA design is critical for optimizing specificity. To further validate the selection of the best possible *myca* sgRNAs, their potential off-targets effects were also manually evaluated using the NCBI reference genome for *O. niloticus*. Sequences for sgRNA1, sgRNA2, and sgRNA3 were searched against nucleotide sequences using Blastn limited to highly similar sequences (megablast) and restricted to entries associated with the organism “*Oreochromis niloticus*” (taxid:8128). No off-target genes were identified that matched any of the three top scoring *myca* sgRNAs suggested by CRISPOR and SYNTHEGO tools. These top three sgRNAs were cloned into an optimized tilapia sgRNA expression vector, as described previously^32^. Complementary oligonucleotides (Eurofins Genomics) comprising each sgRNA’s forward and reverse sequences (Table S1) were annealed to generate a ClaI restriction site at the 5’ end an XbaI restriction site at the 3’ end. The annealed oligonucleotide was then ligated into ClaI (New England BioLabs) and XbaI (New England BioLabs) double-digested TU6m-gRNAscaffHygroR vector^32^ using T4 DNA ligase (Promega). The resulting sgRNA expression vectors for *myca* sgRNAs 1 – 3 were sequenced (sgRNA_seqP1, Table S1) to confirm successful insertion of sgRNA target sequence.

### Transfection and antibiotic-resistance selection of tilapia Cas9-OmB cells

For each well of a six-well cell culture plate, two micrograms of TU6m-gRNAscaffHygroR vector containing either *myca* sgRNA1, sgRNA2, or sgRNA3 were added to 200 µL Opti-MEM I Reduced Serum Medium (Gibco) and 6 µL ViaFect reagent (Promega) to initiate the formation of transfection complexes. Stabilized transfection complexes yielded after 15 min incubation were then applied to 80% confluent Cas9-OmB cells (P43) by adding the transfection complex solution evenly without any medium change. After 48 h, all medium was removed for transfected cells and a non-transfected control and replaced with 2 mL of L-15 medium containing selection media containing 500 µg/ml hygromycin B (Invitrogen, 10687-010). Transfected wells were maintained in selection medium until one day after all cells were detached from the surface of the non-transfected control well. Half of the wells that were transfected with each *myca* sgRNA were then used for analyzing *myca* ko efficiency. This was done as previously described^32^. Briefly, medium was removed and cells surviving hygromycin B treatment were rinsed with Dulbecco’s phosphate buffered saline (DPBS, Gibco, 14190-144). They were then scraped from the surface of the well into fresh 0.5 mL DPBS, transferred to a 1.5 mL microcentrifuge tube, and centrifuged for 5 min at 18,000 g. After removal of supernatant, cell pellets were lysed in 20 µl of 25 mM NaOH by incubation at 95 $^\circ$C for 15 min followed by addition of 50 μl of 40 mM Tris–HCl. The resulting solution containing extracted template DNA was used directly for PCR to generate *myca* test amplicons for analysis of mutational efficiency.

### Limiting dilution strategy

After selection of hygromycin B-resistant cells containing TU6m-gRNAscaffHygroR vector expressing either *myca* sgRNA1, sgRNA2, or sgRNA3, the genetic heterogeneity of mutated, selected cells was serially reduced using a limiting dilution strategy to generate more homogeneous ko cell lines. The protocol for this strategy was adapted from a previous publication^34^ and public protocols (https://www.synthego.com/resources/Limiting-Dilution-&-Clonal-Expansion-Protocol, https://www.addgene.org/protocols/limiting-dilution/) and modified as follows: Selected ko cells were allowed to recover for 14 days in culture to reach 20–30% confluency. They were then harvested as a single cell suspension, counted with a hemocytometer (Hausser Scientific), and diluted to an average concentration of one cell per well before plating into a 24-well cell culture plate. The wells were visually screened periodically to track cells forming colonies using an inverted microscope (DMi1, Leica) for 14 days. When colonies reached 60–70% confluency, they were harvested and split evenly into two new wells of a six-well plate. One well was used for continuous expansion and the other for genotyping the *myca* mutation in the corresponding cell population.

Another round of serial dilution was performed after genotyping the cell populations resulting from the first round of dilution. Further limiting dilution for isolating a single clonal *myca* ko cell line was performed by splitting the most promising cell population from dilution round 1 (sgRNA1-colony#3, see results and Fig. S3) into a 96-well plate at an average density of one cell per well. Cell density was determined by counting cells in a single cell suspension after harvest with hemocytometer (Hausser Scientific). The cell suspension was then diluted to 5 cells per mL medium. Each well received 100 µL of this cell suspension such that the average seeding density was 0.5 cells/well. Seeding an average of 0.5 cells/well ensures that some wells receive a single cell, while minimizing the likelihood that any well receives more than one cell. Cells were maintained for 14 days to track the wells containing a single clonal cell by regular inspection with a microscope (DMi1, Leica).

### Genotyping

Genomic DNA was extracted using PureLink Genomic DNA mini Kit (Invitrogen) following manufacturer instructions. The test amplicon spanning the targeted region of *myca* was PCR-amplified using appropriate primers (Primer pair: myca_TideF1 and myca_TideR2, Table S1) and purified. Sanger sequencing was carried out at the DNA Sequencing Facility of UC Davis using the same primers as those used for PCR. DNA sequences and chromatograms were then analyzed with TIDE (Tracking of Indels by Decomposition; shinyapps.datacurators.nl/tide/) and ICE (inference of CRISPR Edits; SYNTHEGO—CRISPR Performance Analysis) to obtain quantitative overall target gene editing efficiency and indel mutation frequencies from each mono- or poly-clonal *myca* ko cell line. The PCR amplicon using genomic DNA extracted from wild-type Cas9-OmB cells was used as the control sample.

### Cellular phenotyping

Cells were seeded in 6-well plates (Corning, Tewksbury, MA, USA) containing 2 mL complete L-15 medium. After 72 h, attached and proliferating cells were visualized by phase contrast microscopy using an inverted microscope (DMi8, Leica).

### Prediction of 3D protein structure

To confirm functional ko of the resulting truncated protein the structure of *myca* ko truncated protein was compared to wild-type protein. Geneious 2022.0.1 (Biomatters, https://www.geneious.com) bioinformatics software was used to predict pre-mature translation termination (early stop codon) resulting fromdeletion of a single nucleotide from wild-type *myca*in a monoclonal ko mutant (*myca* ko clonal (-1), see results). The resulting mutant MYC protein sequence was generated by translating the cDNA sequence and compared to the wild-type MYC protein sequence. Both (mutant and wild-type) MYC sequences were annotated with functional domains using InterPro version 90.0 (EMBL-EBI)^44^. Moreover, the 3D protein structures were predicted for both (mutant and wild-type) MYC proteins and visualized using AlphaFold^45^,Mol*3D Viewer^46^, and RCSB Protein Data Bank^47^. Combined with the above prediction tools, FunFOLDQA^48^, a protein ligand binding site residue prediction tool, was also employed to reveal whether any DNA binding capacity is preserved in the truncated mutant MYC protein.

## Results

### Identification of evolutionarily conserved MYC domains for functional inactivation

**Figure 1:**
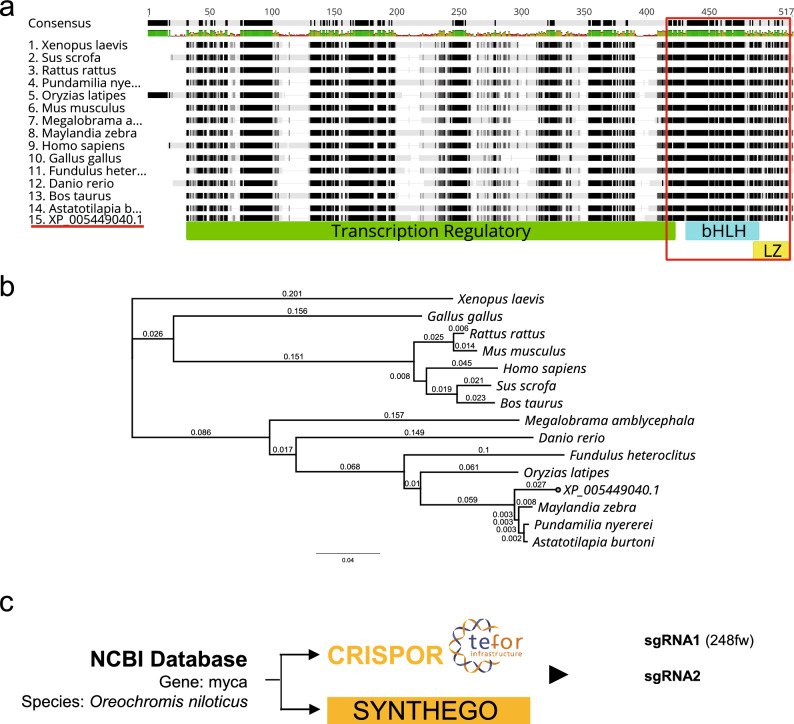
Evolutionary conservation of MYC protein domains and location of *myca* sgRNAs for CRIPSR/Cas9-mediated gene-editing. (**a**) Multiple alignment of MYC protein sequences from 15 vertebrate species including mammals, amphibians, and fishes generated with Geneious (Biomatters, cost matrix = Blosum62). The names of species included in the alignment are listed at the left side. The Oreochromis niloticus MYC protein sequence is labeled by its NCBI accession number (XP_005449040.1) and underlined red. Black blocks indicate highly conserved regions while lighter ones (grey blocks) represent less conserved regions. The transcriptional regulatory domain is depicted by a green-colored bar and the DNA binding domain (bHLH plus LZ) is depicted by cyan-colored bars. The pairwise identity of the DNA binding domain is outlined in red. (**b**) Phylogenetic tree corresponding to the alignment shown in panel a generated using Geneious (Biomatters, genetic distance model = Jukes-Cantor, method = Neighbor-Joining). (**c**) Workflow of sgRNA design using the *O. niloticus* NCBI reference genome to screen for off-target effects identified the three best sgRNAs for tilapia myca.

MYC TF orthologs are highly conserved across many species although the N-terminal part is often more variable than the C-terminal part of MYC, indicating that the latter has been functionally more highly conserved during evolution^6^. In addition to its canonical cellular functions, MYC (encoded by *myca*) may contribute to tilapia osmoregulation, as *myca* mRNA is induced during hyperosmolality and multiple MYC binding sites (E boxes) have been identified in the promoter region of the highly hyperosmotically induced tilapia gene *IMPA1.1* (Supplementary Fig. 1). To identify the domains that are important for tilapia MYC function and ensure that all functionally important domains are inactivated by CRISPR/Cas9 gene targeting, the tilapia *myca* gene (Gene ID: 100689989) was identified in the NCBI reference genome sequence for *Oreochromis niloticus* (NC_031974.2), along with the corresponding mRNA (XM_005448983.4) and protein (XP_005449040.1) sequences. The 432 amino acid sequence of tilapia MYC TF was then used to annotate functional domains using InterPro version 90.0^44^ (EMBL-EBI). This approach identified the long transcriptional regulatory domain at N-terminus and the short DNA-binding domain, consisting of basic helix-loop-helix (bHLH) and leucine zipper (LZ) motifs, at C-terminus (Fig. 1a,d).

To identify the evolutionarily most highly conserved regions in these functional domains, the tilapia MYC protein sequence was aligned to MYC sequences from 14 other vertebrate species. The transcriptional regulatory domain was not found conserved as a whole but rather contained multiple regions that were more highly conserved and interspersed between more divergent stretches of amino acid sequence (Fig. 1a). In contrast, the entire DNA-binding domain, especially the bHLH part of this domain, was highly evolutionarily conserved with a pairwise identity of 90.8% among all 15 species. A phylogenetic tree generated with Geneious (Biomatters) based on the multiple sequence alignment illustrates that tilapia MYC is most similar to MYC of other African cichlids, followed by other euryhaline teleosts (medaka and killifish) (Fig. 1b).

Because highly conserved clusters of sequences were found in the N-terminal transcriptional regulatory domain of tilapia MYC, we aimed to design sgRNAs in the very beginning of the coding sequence, to avoid retention of any potentially functional domain in a truncated mutant protein resulting from frame-shift mutation. Two sgRNAs—**sgRNA2** (rk#3) and **sgRNA3** (rk#4)—designed with CRIPSPOR and SYNTHEGO both met this criterion, in addition to having the lowest predicted off-target effects. A third sgRNAs—**sgRNA1** (248fw) – had the highest scores in both CRISPOR and SYNTHEGO, but was located in the earlier part of the transcriptional regulatory domain (227 bp downstream of the start codon), which means that the truncated mutant protein produced by this sgRNA still contains two highly conserved sequence blocks of the transcriptional regulatory domain (Fig. 1c). PCR primers for amplifying a region that includes target loci of all three sgRNAs (test amplicon) were designed and subsequently used to sequence mutated genomic loci. This test amplicon was sequenced for the wild-type *O. mossambicus* Cas9-OmB cells and compared to the *O. niloticus* reference sequence. The pairwise sequence identity of this test amplicon between *O. mossambicus* and *O. niloticus* was 96.9% overall and 100% for all three sgRNA target sequences (Supplementary Fig. 2).

### Isolation and serial dilution of myca knockout cell lines with low genetic variability

**Figure 2:**
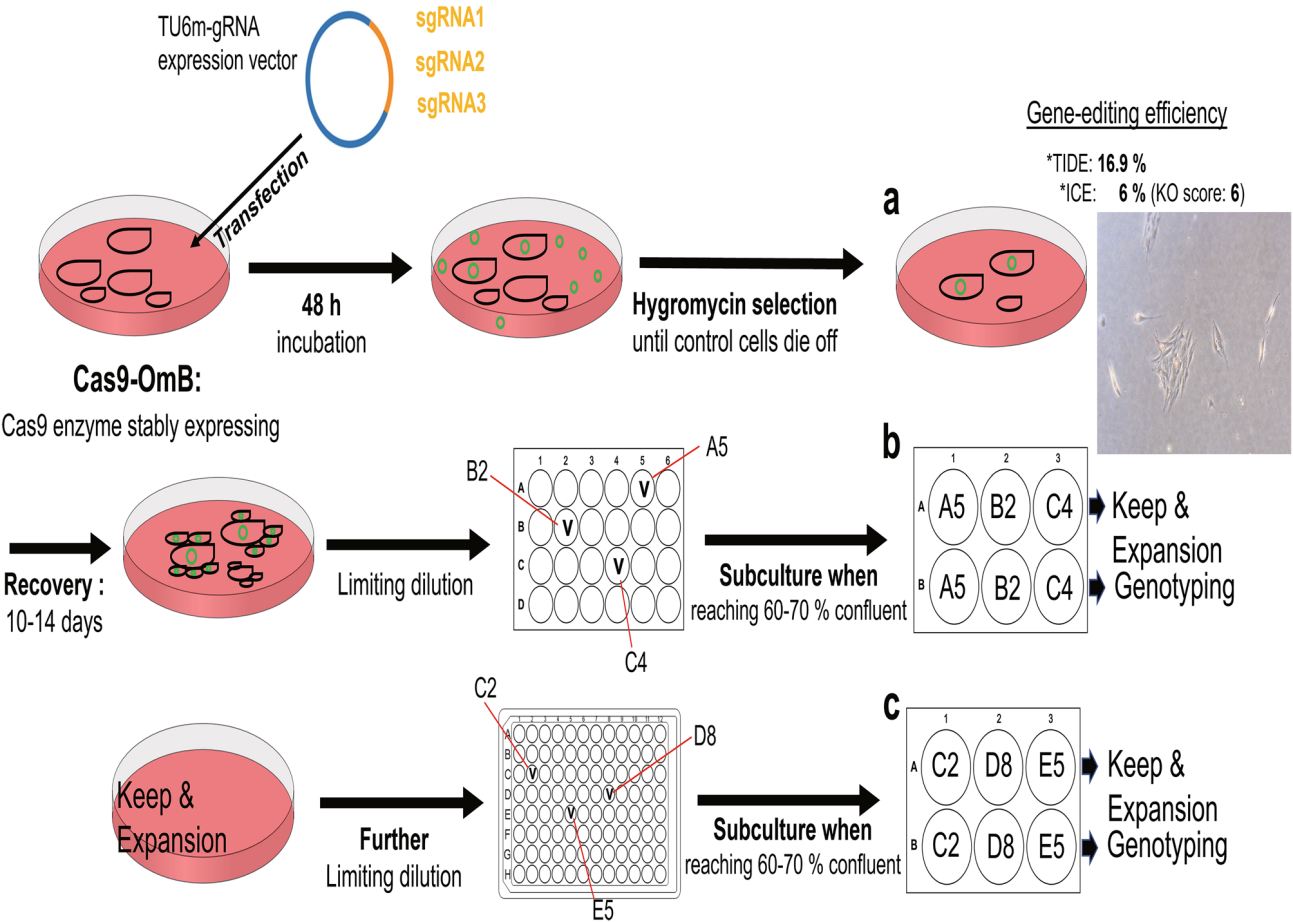
Schematic procedure of initial sgRNA-expression vector transfection (**a**) followed by antibiotic reagent selection and limiting dilution strategy to enrich and isolate poly-clonal cell pools (**b**) and monoclonal cell lines (**c**). Blue circle depicts sgRNA expression vector containing each sgRNA (orange part in the vector) targeting the myca gene. Cas9-OmB cells are depicted as black cell-shaped components in dishes. Green small circles in cell culture dishes represent plasmid vector used for transfection. (**a**) Representation of the initial Hygromycin-selection of highly heterogeneous myca ko cells. The micrograph at the right illustrates the very low confluency of cells surviving the selection process. CRISPR/Cas9-mediated gene editing efficiency of the initial batch of selected cells is low as indicated by TIDE (Tracking of Indels by Decomposition; shinyapps.datacurators.nl/tide/) and ICE (Inference of CRISPR Edits; SYNTHEGO—CRISPR Performance Analysis) scores above the cell micrograph. The Knockout (KO)-score is even lower (6%) indicating that the proportion of cells with a functional ko is very low. (**b**) Initial series of limiting dilution after recovery of the initially selected cells shown in (**a**). The wells marked with ‘v’ (A5, B2, and C4) represent wells containing cell colonies. When these cell colonies reached 70% confluency, they were split 1:2 and transferred to two new wells, one of which was propagated for expansion and the other harvested for genotyping. (**c**) Second limiting dilution series using a single polyclonal ko cell pool generated in (**b**). Each well of the 6-well plate is labeled with the well locations (C2, D8, and E5) of the previous step. A ‘v’ marks wells that contained cell colonies (C2, D8, and D5). These were grown to 70% confluency, split 1:2, and transferred to two new wells, one of which was propagated for expansion and the other harvested for genotyping.

The first *myca* gene editing experiment was performed with sgRNA1. In this pilot experiment, the sequence of the target locus in Cas9-OmB cells transfected with TU6m-sgRNA1-expression vector and selected with hygromycin was compared to that of wild-type Cas9-OmB control cells at the same locus immediately after hygromycin selection. The Sanger sequencing chromatograms of wild-type and sgRNA1 mutant *myca* test amplicons as analyzed by TIDE and ICE tools indicated relatively poor gene editing efficiency of 16.9% (TIDE) and 6% (ICE) (Fig. 2a).

To improve the gene editing efficiency of selected cells, isolate cells with biallelic *myca* ko, and eliminate genetic heterogeneity of isolated mutant cell populations, a limiting dilution strategy was employed in a series of experiments that utilized all three *myca* sgRNAs. In these experiments, cells were allowed to recover from selection by incubation in complete media for 14 days. Cell recovery restored proliferation rate and provided sufficient cell numbers for applying a strategy of limiting dilution. Dilution of the initial *myca* ko cell mixture into a 24-well plate at an average density of one cell per well (Fig. 2b), dramatically improved the overall gene editing efficiency scores in the resulting cell lines ranging from 46.8% to 98.4% in TIDE efficiency and from 37 to 92% in ICE efficiency. The highest gene editing efficiency was 98.4% (TIDE) and 92% (ICE) for sgRNA1 colony #4 (Table S2).

In addition to indel percentage, ICE analysis also provides a KO-score that is derived from calculating the proportion of indels that cause a frameshift or are longer than 21 bp. For instance, the KO-score of sgRNA1-colony#4 was 42/100. This score suggested that, although virtually all cells in sgRNA1-colony#4 had been mutated, less than half yielded a functionally severely impaired protein since 58% did not harbor a frameshift mutation or a deletion of more than seven amino acids.

**Figure 3:**
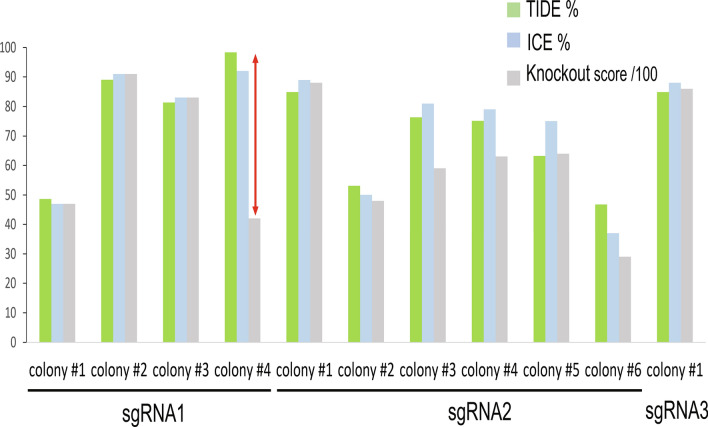
Quantitative indel mutation efficiencies of cell colonies (pools) produced by limiting dilution of cells transfected with sgRNA1, sgRNA2, and sgRNA3 plasmids. Individual cell colonies isolated by the limiting dilution method into individual wells of a 24-well plate at an average density of one cell per well were genotyped and quantitatively analyzed using both TIDE and ICE tools. In addition to obtaining indel mutational efficiencies, ICE provides a KO-score which is the proportion of indels with a frameshift or exceeding 21 bp in length. The resulting bar-graph visually indicates high consistency of all scores except for sgRNA1-colony#4, which has a lower KO-score.

Nevertheless, such discrepancy between gene editing efficiency and KO-score was the exception and the two scores were virtually identical for most mutant cell populations isolated after limiting dilution. For example, sgRNA1-colony#1 had a TIDE score of 48.6%, ICE score of 47%, and a KO-score of 47/100. sgRNA1-colony#2 had a TIDE score of 89.4%, ICE score of 91%, and a KO-score of 91/100. sgRNA2-colony#1 had a TIDE score of 84.9%, ICE score of 89%, and a KO-score of 88/100. sgRNA2-colony#4 had a TIDE score of 53%, ICE score of 50%, and a KO-score of 48/100 (Table S2). Thus, the majority of indel mutations that were isolated and enriched by the limiting dilution strategy resulted in effective frameshifts and potentially severe MYC truncation (Fig. 3).

The 1-bp deletion genotype present in the knockout pool of sgRNA1-colony#3 showed the highest contribution (57%) of any single genotype in any of the cell populations isolated by the first limited dilution series (Supplementary Fig. 3). This result suggested that sgRNA1-colony#3 was the most promising for isolating a monoclonal mutant cell line harboring a *myca* frameshift ko. Thus, we decided to perform a second series of limiting dilution for this population of cells after generating a single cell suspension of sgRNA1-colony#3 by trypsinization of those cells and passing them several times through a serological pipet (described in Fig. 2c).

**Figure 4:**
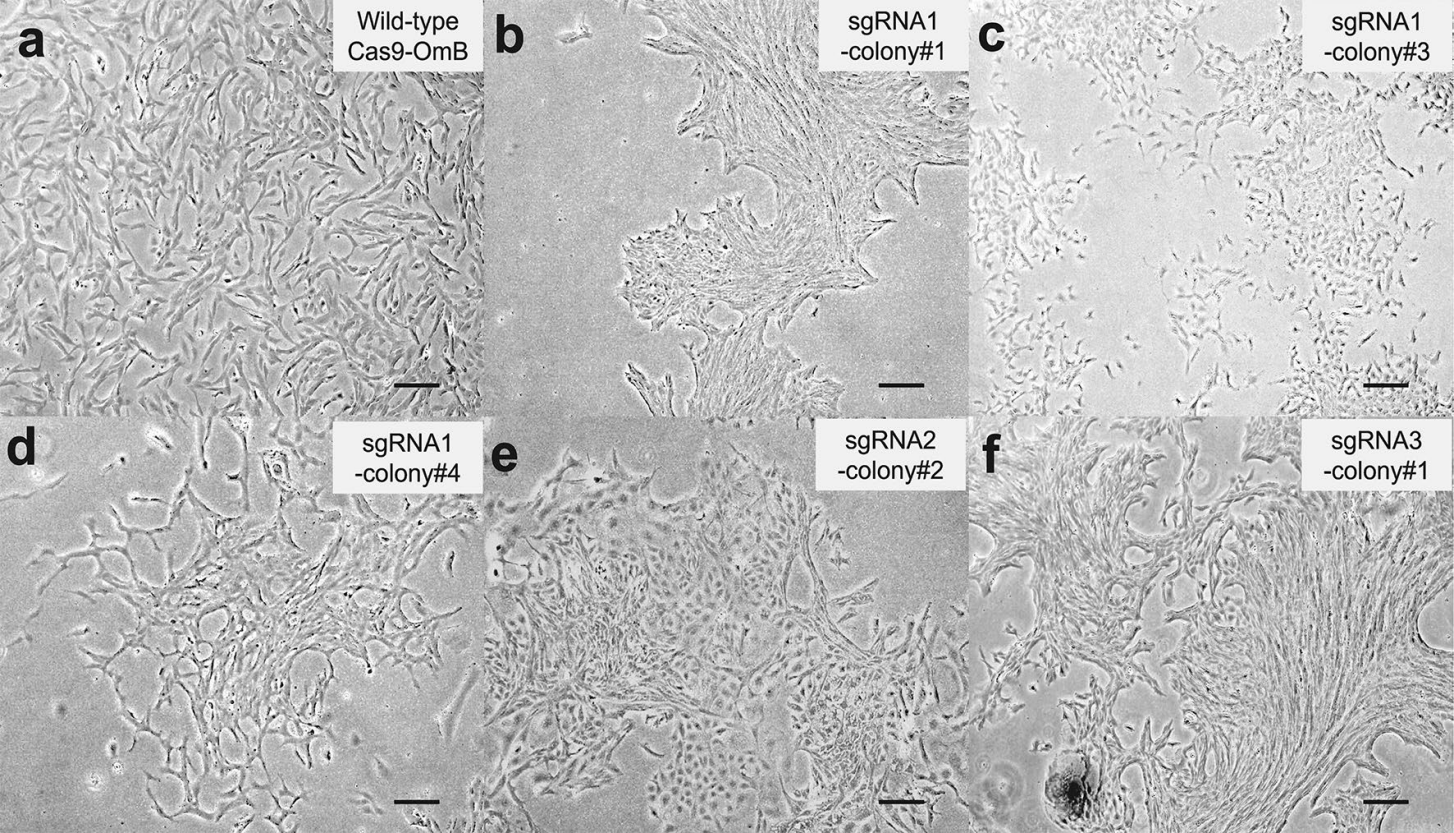
Representative images of cell morphology of wild-type Cas9-OmB cells and *myca* polyclonal ko cell pools (Scale bar, 200 μm). All micrographs were taken on an inverted microscope (Leica Dmi8) and imaged 5–7 days after transferring the cell colonies grown and tracked in a 24-well plate to a 6-well plate.

Altered morphologies and growth patterns were observed in most *myca* ko cell pools resulting from the first series of limiting dilution. For example, *myca* ko cells appeared more adherent to each other (Fig. 4b), had altered growth patterns resulting in more densely clustered patches (Fig. 4e,f), and had lower proliferation rates than wildtype cells, i.e., they need more time to reach confluency (e.g., Fig. 4a,d).

### Isolation of a myca-knockout monoclonal tilapia cell line

The first series of limiting dilution produced polyclonal ko cell pools with high gene editing efficiency that can be directly used for functional analyses. However, to unambiguously rule out any off-target effects on the phenotype of interest it is preferable to use multiple homogenous monoclonal ko lines for functional analyses. The statistical likelihood that two different monoclonal lines harbor the same off-target mutation is infinitesimally small. Thus, if a consistent phenotype is observed it cannot be due to off-target effects.

Therefore, the possibility of generating a monoclonal ko line by another series of limiting dilution of ko cells was explored. To demonstrate proof of principle the sgRNA1-colony#3 ko cell pool resulting from the first limiting dilution series was chosen because it included a 1-bp deletion genotype that accounted for 57% of the total cell population (Supplementary Fig. 3).

**Figure 5:**
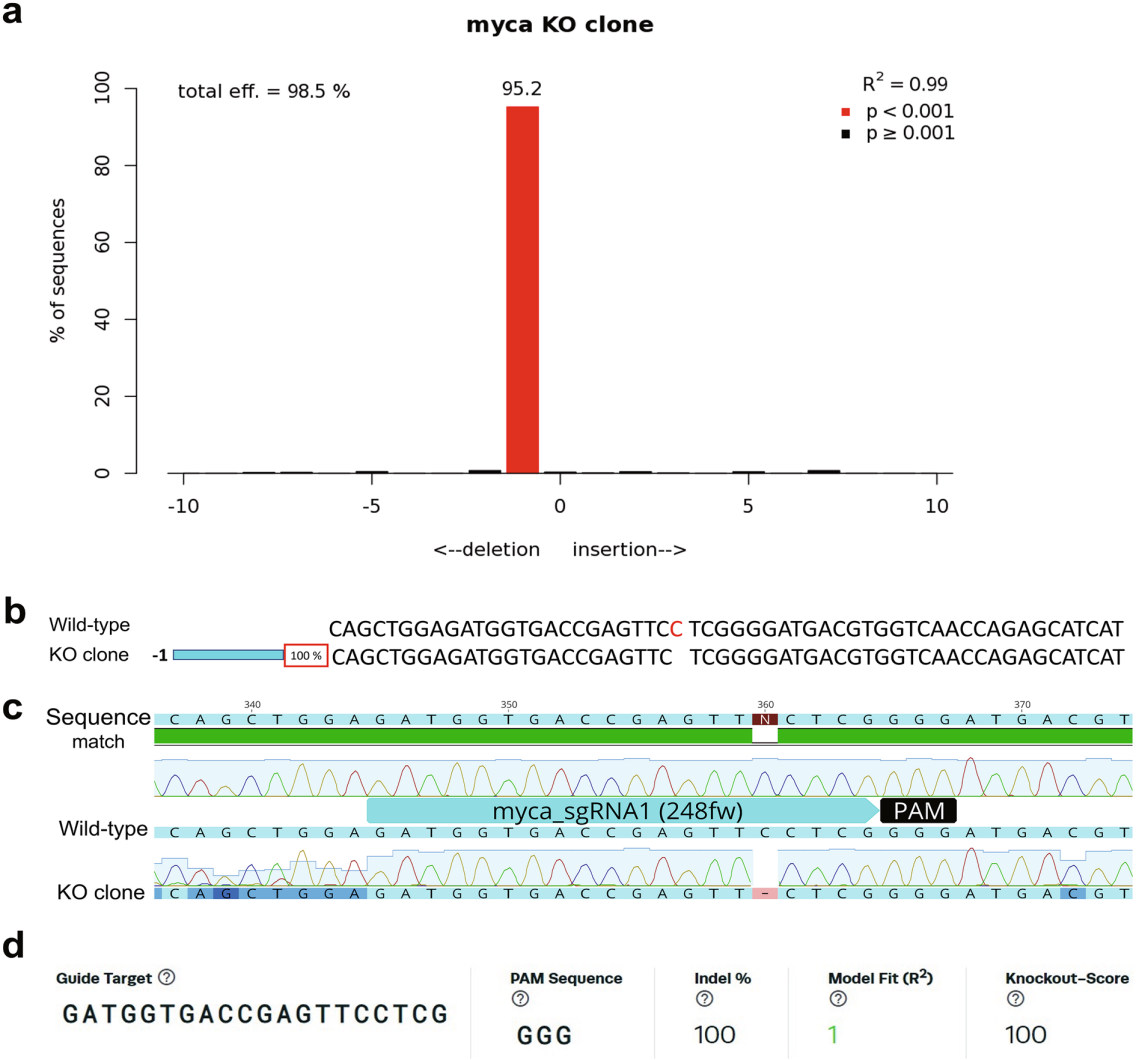
Confirmation of the homogeneity of mutant genotype in a *myca* monoclonal ko cell line. (**a**) TIDE analysis of genomic DNA extracted from a cell colony after the second series of limiting dilution. The X-axis indicates the nature of indels while the Y-axis depicts the percentages of the corresponding sequences. R-square refers to quality of the sequence reads from Sanger sequencing chromatograms with a value above 0.9 considered acceptable. The significance cutoff was set at a default p < 0.001 threshold. (**b**) The ICE analysis result of sequence distribution and frequency (%) of myca monoclonal ko cell line. The top row of nucleotides shows the wild-type sequence for the region surrounding target site. The bottom row indicates the gene-edited mutant sequence (ko clone). The dashed vertical black line indicates the cut site. Both sequences are aligned perfectly except for the 1-bp deletion in the mutant. (**c**) Alignment of wild-type and gene-edited (ko clone) sequencing chromatograms produced with Geneious 2022.0.1 (Biomatters). The cyan-colored bar depicts sgRNA1 target sequence and the black bar shows PAM sequence. (**d**) ICE results include sgRNA target sequence, PAM sequence, indel efficiency, model fitness (R^2^), and Knockout-Score.

This cell pool was diluted and seeded into a 96-well plate at an average concentration of 0.5 cells per well. Two cell colonies were detected after 14 days of seeding and isolated into separate wells. One of these colonies was confirmed to be monoclonal. It consisted of the 1-bp deletion genotype that was most abundant in the starting population of cells. TIDE analysis showed 98.5% gene editing efficiency with R^2^ = 0.99 (Fig. 5a), and ICE indel efficiency was 100% with R^2^ = 1 (Fig. 5d). Moreover, the KO-score was 100/100, which indicated that the 1-bp deletion mutation in the *myca* gene might result in functional disruption of MYC TF. ICE analysis confirmed a homogeneous monoclonal genotype consisting to 100% of the 1-bp deletion mutant (Fig. 5b). The original Sanger sequence trace also confirmed cleanly that the 1-bp deletion of a cytosine was present in all copies of the target test amplicon (Fig. 5c). These data provide evidence that a two-step serial limiting strategy can be applied for isolating monoclonal ko mutant cell colonies from an initially highly heterogeneous mixture of genotypes.

### Structure of the predicted loss-of-function monoclonal mutant MYC protein

**Figure 6:**
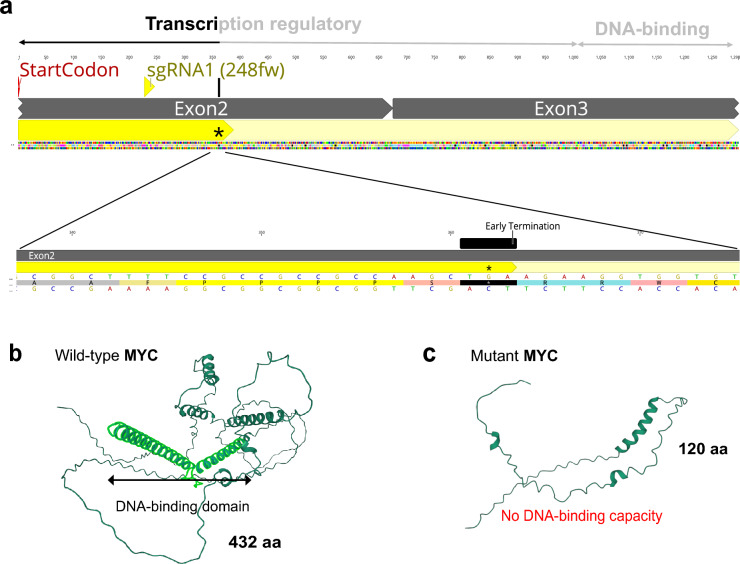
Prediction of loss of function due to truncation of a mutant MYC protein produced by the monoclonal Cas9-OmB-mycaKO1 cell line. (**a**) Wild-type and mutant MYC amino acid sequences were annotated on the coding sequence (CDS, shown yellow highlighted in the upper row). The region of the mutant MYC sequence that is delimited by a premature stop codon (TGA) caused by a 1 bp deletion in the target site is enlarged in the bottom row. (**b**) 3D structure of wild-type MYC protein (432 amino acids) as modeled using AlphaFold (https://alphafold.ebi.ac.uk/entry/Q45RH2), Mol*3D Viewer, and RCSB Protein Data Bank. The scissor-like structure composed of two alpha-helixes (light green) represents the main DNA-binding domain. (**c**) 3D structure of mutant MYC protein (120 amino acids) as predicted using the same tools and approach as in panel (**b**).

Compared to wild-type MYC protein made up of 432 amino acids, the mutant MYC protein expressed in the monoclonal OmB-*myca*KO1 mutant cell line was predicted to be truncated to only contain the first 120 amino acids due to premature translation termination via an early stop codon (TGA) (Fig. 6a). The majority of transcription regulatory domain and all of the DNA-binding domain (bHLH + LZ) were missing in the mutant MYC protein, indicating elimination of its function as a TF.

Protein structures of both wild-type and mutant MYC as generated using AlphaFold^45^, RCSB Protein Data Bank^47^, and Mol* 3D Viewer^46^ were profoundly different between wild-type and mutant MYC (Fig. 6b,c). The removal of protein domains necessary for TF function from the truncated mutant protein was confirmed using FunFOLDQA^48^, a protein ligand binding site residue prediction tool, which indicated ‘No DNA binding capacity’ of the truncated mutant MYC protein.

### Morphological differences between Cas9-OmB-mycaKO1 and wildtype Cas9-OmB cells

**Figure 7:**
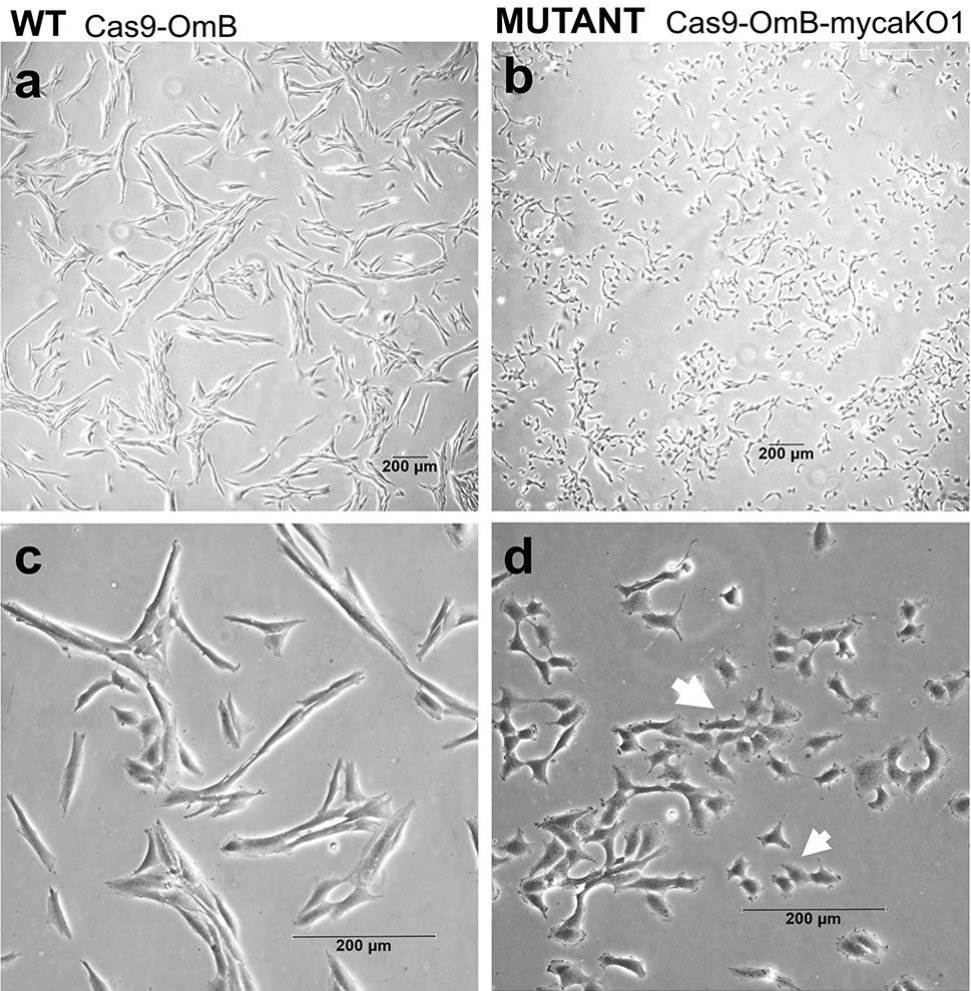
Cell morphology of tilapia cell lines. Micrographs showing differences in morphology of Cas9-OmB wild-type cells (**a**,**c**) versus Cas9-OmB-mycaKO1 mutant cells (**b**,**d**). The lack of elongated cell extensions in selected mutant cells is illustrated by white arrows. Images were taken using 5 × and 20 × phase objective on an inverted microscope (Leica Dmi8). The bar at the bottom of each image indicates a distance of 200 μm.

In addition to the general tendency for forming tighter cell clusters and decreased proliferation rate (time to confluency) outlined for heterogeneous mutant cell populations above, a conspicuous morphological difference was observed for the Cas9-OmB-*myca*KO1 cell line compared to *myca* wild-type Cas9-OmB cells. The morphology of both wild-type and mutant cell lines was compared after two additional passages to expand the mutant line for cryopreservation. The mutant cells appeared smaller due to much shorter cell extensions (Fig. 7, white arrows) compared to in the morphology of wildtype cells showing elongated cell extensions even though the main cell body of mutant cells was comparable in size to wild-type cells (Fig. 7).

## Discussion

### Potential non-canonical role of MYC as an osmoregulatory transcription factor

MYC is a well-studied transcription factor having numerous cellular functions such as the regulation of cell growth, cell cycle, cell differentiation, global mRNA translation, and cellular stress response (CSR) in a wide variety of organisms^49–51^. MYC has been studied extensively in the context of cancer biology, because of its overexpression in malignant tumors and activation of many hallmarks of cancer^52^. However, other functions of MYC that are not directly relevant for cancer biology have not received much attention. Since MYC TF is evolutionarily highly conserved and have many transcriptional targets^5^, they are likely central regulators of the CSR and other diverse cellular functions that are important for normal non-pathological physiology^53^.

To enable causal links between MYC function and the CSR in tilapia, in particular during salinity (osmotic) stress, this study generated tilapia *myca* ko cell lines. Previous studies suggest that *myca,* which is the gene encoding for tilapia MYC, may be involved in governing mechanisms of teleost osmoregulation^8,13^. Furthermore, our results show that, unlike *myc2*, *myca* is elevated at the mRNA level during hyperosmotic stress in tilapia OmB cells (Supplementary Fig. 1a). MYC regulates its downstream target genes by binding to the E-box (CACGTG or CATGTG), a MYC-specific cis-regulatory element (CRE).

Intriguingly, three E-box sequences were found in the proximal promoter region (within 1.1 kb of the transcription start site) of the tilapia *IMPA1.1* gene encoding the most osmoresponsive enzyme in tilapia (Supplementary Fig. 1b). We had previously shown that hyperosmotic transcriptional induction of *IMPA1.1* is at least partly mediated by several osmolality/salinity-responsive element 1 (OSRE1) CREs^54^. The OSRE1 core consensus sequence resembles that of the mammalian tonicity response element (TonE), which is the binding site for nuclear factor of activated T-cells 5 (NFAT5)^55^. This similarity suggests that tilapia NFAT5 contributes to the osmotic regulation of the *IMPA1.1* gene.

However, gene expression is often regulated in a combinatorial manner and depends on multiple different CREs and TFs. Our findings of hyperosmotic MYC TF mRNA elevation and the presence of multiple MYC CREs (E-boxes) in *IMPA1.1* suggests that combinatorial transcriptional regulation involving NFAT5 and MYC controls the hyperosmotic induction of tilapia *IMPA1.1*. To enable functional analyses of the role of MYC and its interaction with NFAT5 for osmoregulatory target gene transactivation this study generated multiple poly- and mono-clonal *myca* ko tilapia cell lines that can be used in combination with reporter assays^56,57^, molecular and cellular phenotyping^58–60^, and other approaches^61,62^ to comprehensively characterize the role of MYC for teleost osmoregulation.

### Limiting dilution is an effective strategy for cloning specific cell genotypes

Targeting genes in vitro using a CRIPSR/Cas9 system results in a heterogenous pool of edited cells with varying indel populations^34^. Such highly heterogeneous polyclonal cell pools can have poor overall gene editing efficiency due to inefficient delivery or low sgRNA-dependent mutational efficiency^63,64^. Moreover, some of the cells in this heterogeneous pool may harbor non-specific mutations that can cause off-target effects. One way to eliminate such potential off-targets is to isolate genetically distinct cell pools and check for consistent phenotypes when multiple of these distinct pools are used for experiments that evaluate functional consequences of gene ko. In this study, we applied a serial limiting dilution strategy to increase gene editing efficiency and decrease the genetic heterogeneity of polyclonal and monoclonal mutant (gene-edited ko) cells. Both polyclonal and monoclonal cell lines are useful depending on the purpose of the subsequent experiment^29,36,39^.

We successfully produced ten polyclonal cell pools with reduced heterogeneity in the indel genotypes when compared to the original mixture of gene edited cells. Five of these polyclonal lines had much higher gene editing efficiencies (> 80%). The remaining five cell pools with < 80% gene editing efficiency also showed improved mutational efficiency relative to the starting population. The high gene editing efficiencies achieved during the initial series of limiting dilution illustrate that this strategy represents a rapid way for enrichment of desirable genotypes. However, wildtype mRNA and protein expression from unedited or heterozygous cells harboring a haploid mutant genotype represents a potential pitfall of studying polyclonal ko cells. This would be particularly problematic for long-term studies if unedited or haploid cells have a growth rate that exceeds those of diploid mutants.

To eliminate concerns about possible phenotype masking effects due to the potential presence of unedited or haploid genotypes in a polyclonal cell pool, we performed another series of limiting dilution. For this second dilution series, a polyclonal cell line from the first series of dilution was chosen, which had the highest frequency of frameshift (functionally inactivating) mutations as determined by ICE analysis. We demonstrate that it is possible to isolate a monoclonal tilapia cell line harboring a 1-bp deletion in the *myca* gene after only two rounds of limiting serial dilution. In theory, it is possible to decrease the cell concentration during the first round of dilution even further and seed more aliquots (e.g., into a 1536-well plate) to obtain monoclonal lines after a single limiting dilution step. However, in praxis, our approach of subsequent serial dilutions at an average seeding density of 1 cell per well followed by an average seeding density of 0.5 cells per well yielded the best results with cells retaining their ability to form colonies within a short time (within 4–5 weeks). This approach also reduced the likelihood of seeding cell clusters present in cell suspensions during the second round of limiting dilution^35^. Thus, limiting dilution is an effective strategy to overcome (1) the genetic heterogeneity of mutant cells, (2) the potential presence of unedited or haploid mutant cells in mixed populations, and (3) the low delivery efficiency of vector-based CRISPR/Cas9 in cultured fish cells^65–67^. The limiting dilution strategy is not only fast but also very cost-effective.

Our approach overcomes difficulties often encountered when attempting to isolate clonal mutant cell lines from non-canonical model organisms^68^. For example, workflows for isolating gene-edited clonal cell lines by fluorescent-aided sorting system are currently only feasible for mammalian cells^69^. Moreover, even if expensive cell sorting devices and corresponding labeling approaches are available, cells would be potentially exposed to the non-sterile conditions and in danger of being contaminated. Cell viability is also negatively affected by the cell sorting process. The serial limiting dilution strategy used in this study provides a robust, rapid, and cost-effective platform for generating ko clonal cell lines for studies of mutagenesis effects on cellular phenotypes of teleost fishes.

### Interpretation of gene editing results by TIDE and ICE analyses

To analyze results from CRISPR/Cas9 gene editing experiments, the test amplicon sequence spanning the target site must be analyzed with bioinformatics tools that deconvolute the indel heterogeneity into interpretable scores. We used TIDE and ICE analyses for this purpose. Sanger sequence chromatograms of test amplicons from genomic DNA of gene edited and wildtype cells and the corresponding sgRNA sequence were used as the input data. TIDE^70^ has been widely adopted for mutation detection since its development in 2014 as an accurate, versatile, and time-saving alternative to restriction enzyme-based assays^63,71^. The ICE analysis pipeline (SYNTHEGO)^72^ has been developed more recently and examined rigorously by comparing to NGS-based amplicon sequencing data. This evaluation revealed that the accuracy of ICE analysis is comparable to that of NGS-based approaches such as CRISPResso2, which aligns deep sequencing reads to a reference sequence^73^.

In addition to scoring the overall gene editing efficiency, ICE also provides a Knockout score (KO-score) which is a useful measure to determine how many of the contributing indels are likely to result in a functional ko of the targeted gene. The main advantages of using both TIDE and ICE in combination are being able to: 1. Compare the consistency of two independent measures for gene editing efficiency, which are derived from different analytical algorithms, 2. Obtain detailed information about distribution and frequency of different types of indels, and 3. Estimate the frequency of indels resulting in a functional ko. Because of these advantages and their complementary features, the combined use of TIDE and ICE for indel heterogeneity analyses has been reported previously^74^.

One important finding of our study was a substantial difference between KO-score and indel efficiency observed in the sgRNA1-colony#4. While TIDE and ICE indel efficiency scores for this cell population were 98.4% and 92%, respectively, the KO-score was only 42% (Fig. 3 and Table S2). This information represents a critical factor for choosing particular cell populations for functional studies. In this case, the sgRNA1-colony#4 cells would be eliminated from consideration for functional phenotype analyses because almost half of the mutants generated produced wildtype MYC proteins that is functionally intact. The genetic heterogeneity was higher for other mutant populations including sgRNA2-colonies #2 and #3, which is why sgRNA1-colony#3 was chosen for serial dilution (see also supplementary Fig. 3b).

The scores provided by TIDE and ICE analyses provide effective and unbiased selection criteria of choosing a particular population of cells from the first round of limiting dilution for subsequent serial dilution of cells. We selected the sgRNA1-colony#3 cell population resulting from the first limiting dilution for the second round of limiting dilution because of the high abundance of a specific genotype (a 1-bp deletion indel accounts for 51.4%) and because the frameshift mutation introduced a premature translation termination codon shortly after the target site, Fig. 6). Because of this premature stop codon, the mutant protein that is generated is very short, which minimizes potential non-specific side effects of expressing an abnormal protein in cells that are subjected to complex phenotype analyses. Of note, other colonies (e.g., sgRNA2-colony#2 and #3) with intermedial difference between indel efficiencies (TIDE and ICE) and KO scores (with high indel frequency) were also ruled out for subsequent dilution mainly due to contributions of individual mutant genotypes were less than 51.4% of sgRNA1-colony#3 (Supplementary Fig. 3).

### myca clonal ko tilapia cell line and its potential use and implications

The limiting dilution strategy had already been successfully used in a few cases to generate clonal ko cells in different fish species including carp^39,75^, medaka^76^, and Chinook salmon^38^. These studies were focused on investigating mechanisms of resistance to viral infection. However, generation of polyclonal or monoclonal ko cell lines from heterogeneous indel genotypes generated by CRISPR/Cas9 technology, whether by limiting dilution or other approaches, has not been reported prior to this study for any tilapia species.

Here we established proof of principle that monoclonal tilapia ko cells can be generated by limiting dilution of heterogeneous indel genotypes resulting from using a vector based CRISPR/Cas9 system with cell lines. Such monoclonal ko lines have a defined genotype that facilitates the interpretation of functional studies aimed at evaluating the effects of gene inactivation on cellular phenotypes, for example osmoregulatory, disease resistance, proliferation, or other phenotypes that are informative for understanding basic physiological mechanisms but also of great interest from an applied perspective, e.g. for improving aquaculture^77,78^. Tilapia are widely used aquaculture species, second only to carp regarding global production yields. However, like many other organisms, tilapia are subject to climate change and pollution, which negatively affects their performance and natural habitat and aquaculture. Mechanistic insight derived from gene targeting studies helps to understand, properly interpret, and compensate for such impacts to facilitate mitigating these negative effects.

In the current study, the *myca* gene encoding MYC TF was chosen as to demonstrate the effectiveness of the limiting dilution strategy for generating a clonal ko cell line. This cell line and the polyclonal *myca* ko lines generated in the present study enable testing the role that MYC TF plays for tilapia cellular osmoregulation, its contribution to the activation of the myo-inositol biosynthesis (MIB) pathway, and its contribution to other cell functions. This approach can be extended to other targets and species of interest, for example genes important for aquaculture traits other than salinity tolerance and candidate genes other than *myca*, including those identified by previous GWAS and SNP analyses in multiple aquaculture fish species^79–81^. These ko cell lines allow for deep functional analyses that associate specific genotypes with complex phenotypes, including systems level molecular phenotypes revealed by transcriptomics^82^ and proteomics^83^.

Surprisingly, the cell viability and growth rate of the *myca* clonal ko cell line did not differ from that of wild-type Cas9-OmB cells. This contrasts with several polyclonal ko cell pools obtained after the first round of limiting dilution, which displayed reduced proliferation even after three passages. This result indicates that *myca* ko is not lethal, and even does not hamper cell propagation in vitro. This phenomenon is interesting as we expected that all ko cell lines might show similar reduced cell viability and slower cell growth compared to the wild-type cells because of the known essential roles of MYC in numerous cellular processes including cell growth, proliferation, and differentiation^84^. Nevertheless, the aberrant morphologies of tilapia *myca* ko cell lines (Figs. 4c,d) support the idea that cell differentiation, one key cellular phenotype controlled by MYC TF, is notably altered relative to wild-type cells.

Although aberrant cell morphology is reported for *myca*ko cells in this study, more in-depth molecular phenotyping will help understand the physiological consequences of *myca*ko in future studies. Specifically, osmotolerance phenotypes of *myca* ko cells can be analyzed^42^ and quantitative proteomics approaches can be employed to provide functional insights into biochemical and genetic networks that are controlled by MYC TF^85,86^. The limiting dilution approach and the *myca* ko tilapia cell lines generated in this study will empower such future functional analyses.

In summary, this study has successfully used a vector-based CRISPR/Cas9 approach in combination with a serial limiting dilution strategy to generate mono- and poly-clonal tilapia *myca* ko cell lines for in depth cellular phenotyping studies, directed at investigating functions of MYC TF in tilapia.

## Supplementary Information

Supplementary Information.

## Data Availability

The datasets generated and/or analysed during the current study are available as DNA sequence datafor gRNAsand PCR primers, which are provided in the main manuscript or supplementary data file. The tilapia *myca* gene (Gene ID: 100689989) was identified in the NCBI reference genome sequence for *Oreochromis niloticus* (NC_031974.2), along with the corresponding sequences for mRNA (XM_005448983.4) and protein (XP_005449040.1).
