# Supplementary material for: Removal of evolutionarily conserved functional MYC domains in a tilapia cell line using a vector-based CRISPR/Cas9 system
Source: Sci Rep. 2023 Jul 26;13:12086. doi: 10.1038/s41598-023-37928-x (PMC10371998; doi:10.1038/s41598-023-37928-x)
Supplement: Supplementary file 1 — Supplementary Information. [file 41598_2023_37928_MOESM1_ESM.pdf]

Supplementary Material

Journal: Scientific Reports

Title: Removal of evolutionarily conserved functional MYC domains in a tilapia cell line using a vector-based CRISPR/Cas9 system

Chanhee Kim, Avner Cnaani, and Dietmar Kültz(✉)

Stress-induced Evolution Laboratory, Department of Animal Sciences, University of California, Davis, CA, 95616; dkueltz@ucdavis.edu

Supplementary Figures:

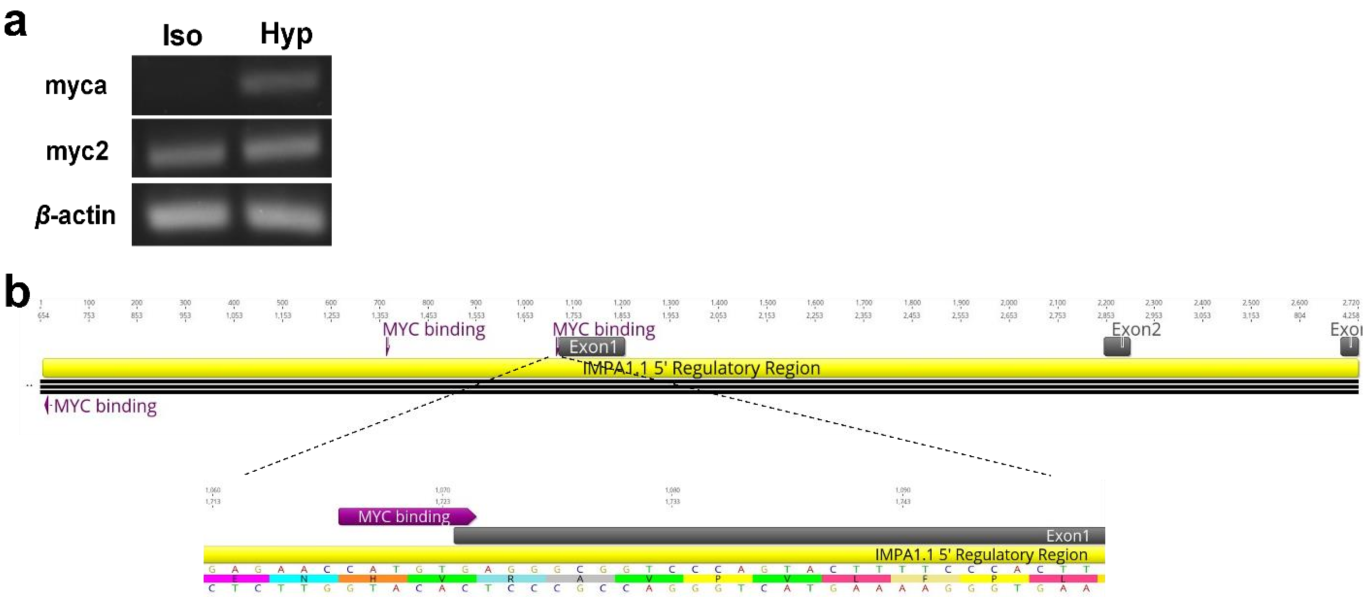

Supplementary Fig. 1 MYC mRNA abundance in tilapia OmB cells. **(a)** Semi-quantitative cDNA agarose gel indicating increased *myca* mRNA abundance after exposure of OmB cells to 650 mOsmol/kg hyperosmolality for 6 h. mRNA was isolated from both iso- (Iso) and hyperosmotically (Hyp) grown OmB cells using PureLink RNA mini kit (Invitrogen, 12183020), reverse transcribed into cDNA using Verso cDNA Synthesis Kit (ThermoFisher, AB1453A), and PCR amplified for 35 cycles using Mastercycler (Eppendorf, 6333000022). **(b)** MYC binding motifs (purple-colored) identified with the Geneious "Find Motifs" function (Geneious 2022.0.1, Biomatters) annotated to the *O. mossambicus IMPA1.1* 5' regulatory region (proximal promoter).

The original (unprocessed) gel image of Fig. S1a

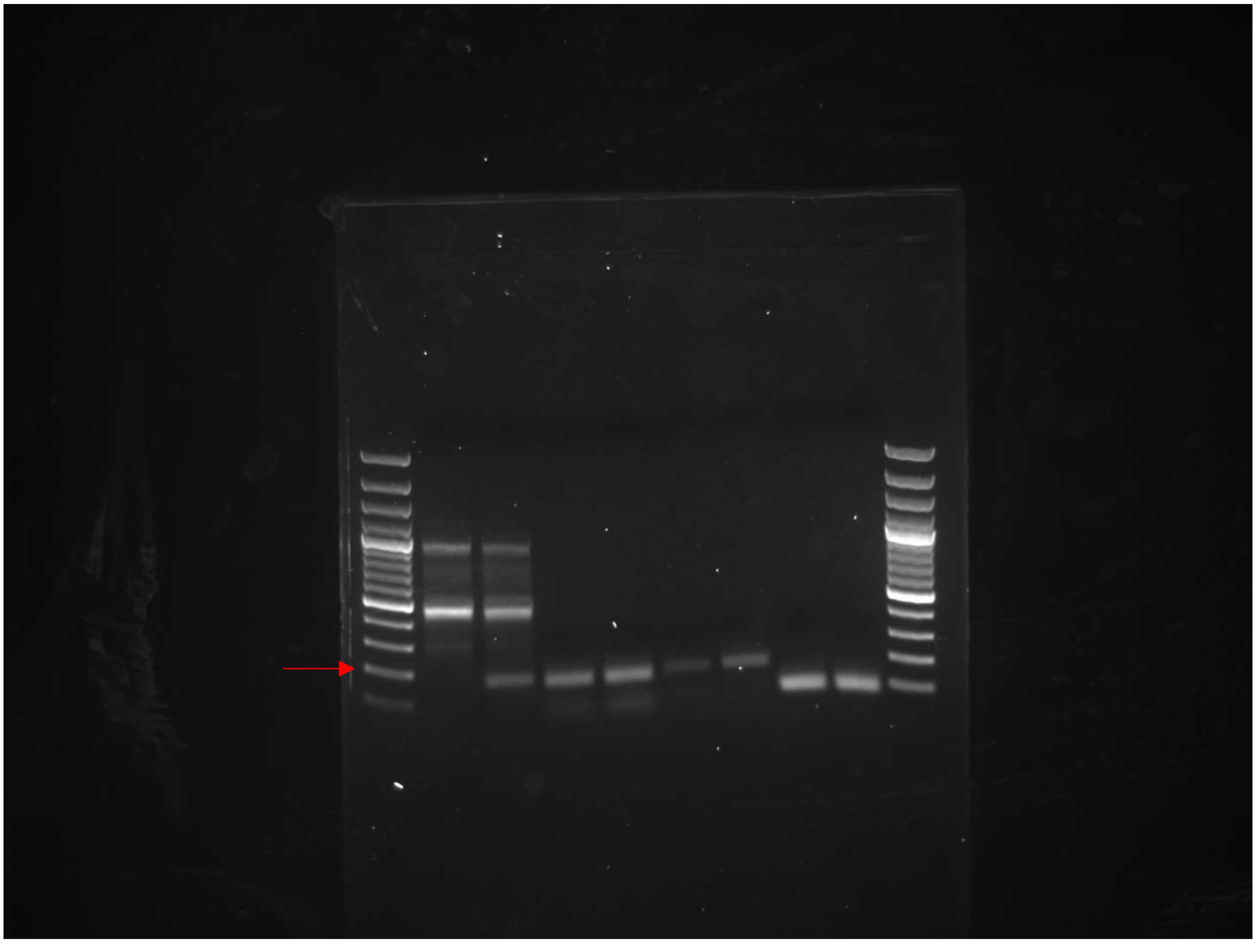

Note: Lane 1 and 10 indicate 100 bp DNA markers. Lane 2: myca (iso), Lane 3: myca (hyp), Lane 4: myc2 (iso), Lane 5: myc2 (hyp), Lane 8: β-actin (iso), and Lane 9: β -actin (hyp). Lane 6 and 7 are loaded with FoxO4 (iso and hyp) transcription factor which is not focused on in this study. iso; isosmotic, hyp; hyperosmotic. The red arrow indicates 200 bp. All targeted transcript sizes range 180-200 bp except for beta-actin transcript which is targeted at 100 bp.

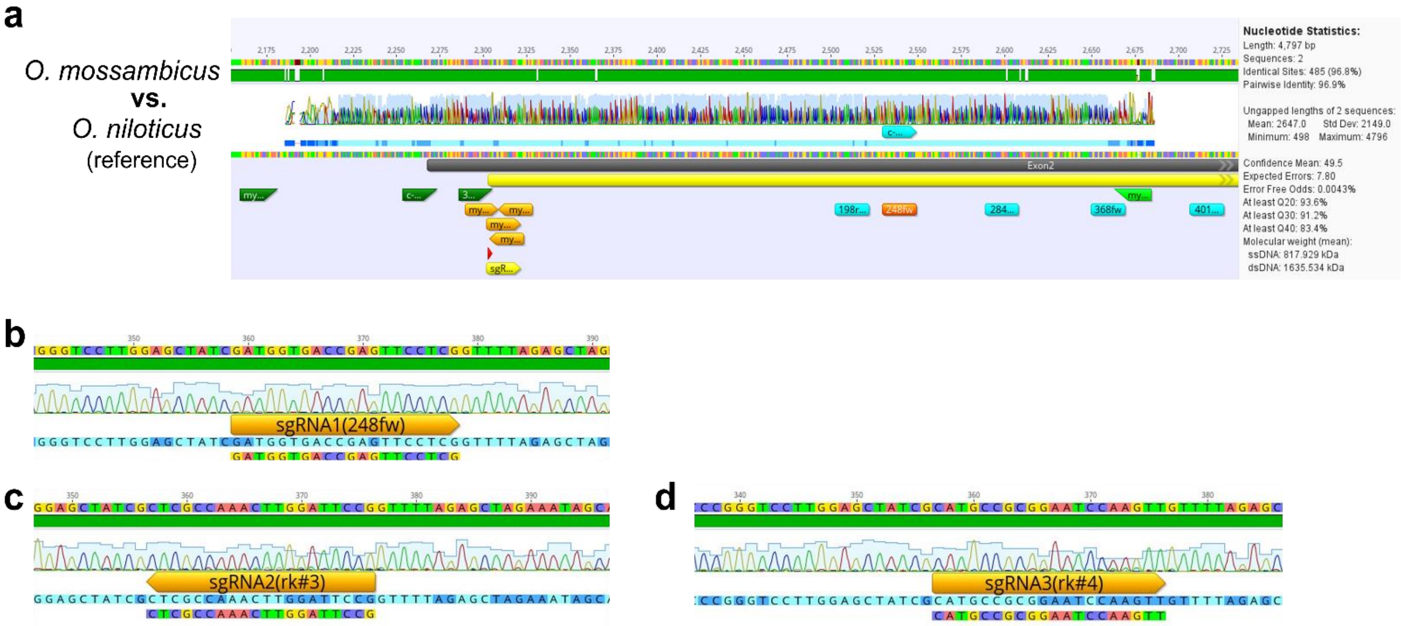

Supplementary Fig. 2 Sequence confirmation of sgRNAs targeting *Oreochromis mossambicus* MYC (*myca*). **(a)** The *myca* targeted sequence (test amplicon region) was compared between *O. mossambicus* and *O. niloticus* by aligning the PCR-amplified sequence covering all sgRNA target sequences with the *O. niloticus* reference sequence. The pairwise identity between the two sequences is 96.9 %. **(b-d)** sgRNA coding sequence validation after cloning into TU6 gRNA expression vector to confirm 100% match of sgRNAs to corresponding *O. mossambicus myca* locus. **(b)** sgRNA1 (248fw) **(c)** sgRNA2 (rk#3), and **(d)** sgRNA3 (rk#4).

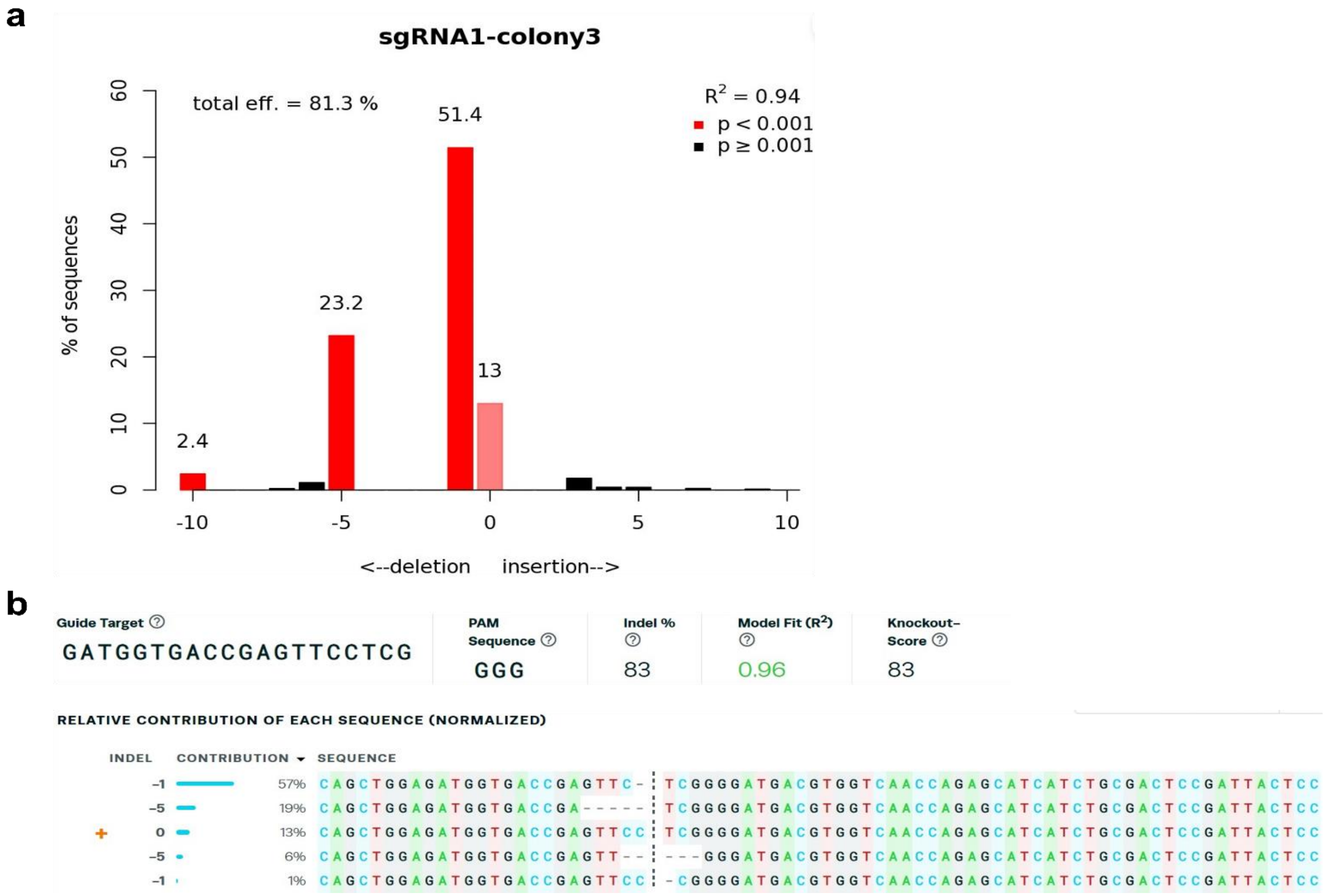

Supplementary Fig. 3 Relative contribution of each genotype present in the sgRNA1-colony#3 ko cell pool after the first round of limiting dilution. **(a)** TIDE analysis result with significant indels shown as red bars (p-value < 0.001). **(b)** Corresponding ICE analysis result showing high agreement with the TIDE data. In addition, ICE analysis also provides the knockout score, which is the same as the Indel frequency (83%). Matching scores for indel frequency and knockout score indicate that all three types of indels present in this cell population (-1del, -5del.1, -5del.2, and -10del) all cause frameshift mutations. The R-square value refers to quality of the sequence reads from Sanger sequencing with a value above 0.9 considered acceptable.

Supplementary Table:

Supplementary Table 1. Sequences of primers used in this study. The bold nucleotides indicate sgRNA targets (5'→3')

Primer Purpose	Primer Name	Primer Sequence
sgRNA oligo annealing	myca_sgRNA1(248fw)Target	CG**ATGGTGACCGAGTTCCTCG**GTTTTAGAG
	myca_sgRNA1(248fw)Compl	CTAGCTCTAAAAC**CGAGGAACTCGGTCACCAT**
	myca_sgRNA2(rk#3)Target	CG**CTCGCCAAACTTGGATTCCG**GTTTTAGAG
	myca_sgRNA2(rk#3)Compl	CTAGCTCTAAAAC**CGGAATCCAAGTTTGGCGAG**
	myca_sgRNA3(rk#4)Target	CG**CATGCCGCGGAATCCAAGTT**GTTTTAGAG
	myca_sgRNA3(rk#4)Compl	CTAGCTCTAAAAC**AACTTGGATTCCGCGGCATG**
Genotyping	myca_TideF1	TGGAGGGAGTTGACCATGAAAG
	myca_TideR2	CTCGGACACCACCTTCTTCA
	sgRNA_segP1 (for sequencing)	GTATACTATGTGCCGAATTTCC
myca mRNA detection	myca_qPCR_F	TGTCACTGCCGCACTGGAAT
	myca_qPCR_R	CAAACTTGGATTCCGCGGCA
myc2 mRNA detection	myc2_qPCR_F	GTGTTCCTGGGTGAGAAGCA
	myc2_qPCR_R	TGTCCACTGTCACCACATCG

Supplementary Table 2. Quantitative indel mutation efficiencies of cell colonies (pools) produced by limiting dilution of cells transfected with sgRNA1, sgRNA2, and sgRNA3 plasmids.

Cell colony #	TIDE efficiency %	ICE Indel %	ICE-KO score /100
sgRNA1-colony#1	48.6	47	47
sgRNA1-colony#2	89.4	91	91
sgRNA1-colony#3	81.3	83	83
sgRNA1-colony#4	98.2	92	42
sgRNA2-colony#1	84.9	89	88
sgRNA2-colony#1	80.6	75	64
sgRNA2-colony#3	74.4	79	63
sgRNA2-colony#4	53	50	48
sgRNA2-colony#5	63.2	75	64
sgRNA2-colony#6	46.8	37	29
sgRNA3-colony#1	84.9	88	86
